# The Ethics Police?: IRBs' Views Concerning Their Power

**DOI:** 10.1371/journal.pone.0028773

**Published:** 2011-12-13

**Authors:** Robert Klitzman

**Affiliations:** Department of Psychiatry, Columbia University, New York, New York, United States of America; Indiana University, United States of America

## Abstract

**Background:**

In recent years, tensions between IRBs and principal investigators (PIs) have risen, posing the needs to understand these conflicts, their underlying causes, and possible solutions. Researchers frequently complain about IRBs, but how IRBs perceive and respond to these criticisms is unclear.

**Methods:**

I conducted in-depth, semi-structured interviews of two hours each with 46 chairs, administrators, and members. I contacted the leadership of 60 IRBs around the country (every fourth one in the list of the top 240 institutions by NIH funding) and interviewed IRB leaders from 34 of these institutions (response rate = 55%).

**Results:**

Interviewees suggest that IRBs and PIs may view the nature and causes of these conflicts very differently and misunderstand each other, exacerbating tensions. Interviewees often recognized that they were seen by PIs as having power, but many IRBs saw themselves as not having it (e.g., because they are “merely following the regulations,” and their process is “open,” impersonal and unbiased, and they are themselves subject to higher administrative agencies), or as having it, but feeling it is small, and/or justified (e.g., because it is based on overriding goals and “the community values,” and IRBs are trying to help PIs). Questions emerge as to whether IRBs do or should have power, and if so, what kind, how much, and when. Several factors may affect these tensions.

**Conclusions:**

This study, the first to explore how IRBs perceive and understand conflicts and power relationships with PIs, suggests how IRBs and PIs may differ in viewing their respective roles and relationships, exacerbating tensions. These issues have critical implications for IRBs and PIs—to enhance their awareness and understanding of these conflicts (e.g., that IRBs may have discretionary power) and the underlying causes involved, and for increasing attention to research, practice, and policy concerning these areas of IRB functioning and interactions with PIs.

## Introduction

In recent years, tensions between institutional review boards (IRBs) and researchers have increased, posing needs to understand these conflicts and their underlying causes, and possible solutions. Many principal Investigators (PIs) have complaints about IRBs [1]–[4], and IRBs have been called the “Ethics Police,” [5] but little is known about how IRBs themselves perceive and respond to these criticisms. Debates persist as to whether IRBs do or should have power, and if so, what kind, how much, when, and why.

IRBs can approve, disapprove, delay, or require changes in studies; and frustrate academic researchers - e.g., because of postponements, and adversarial stances [2], [5]–[6]. Discrepancies have also been documented between IRBs in their decisions [7]–[9]. Some critics have argued that the system is “broken” [10] and that IRBs are even unconstitutional in impeding academic freedom [11].

IRBs have power as gatekeepers [3], and researchers have been found to generally accept the rationale for ethical oversight, but often feel that IRBs focus on unimportant details [1], [2], and are bureaucratic [4]. PIs also tend to value IRBs treating researchers fairly more than protecting subjects per se [3]. PIs who feel they have been unfairly treated by IRBs may be more likely to feel justified in avoiding IRB regulations [12].

In response to criticisms of IRBs by PIs and others, proposals for increased central IRB (CIRB) reviews in multi-site studies have been made for almost two decades [13], [14]. Recently, the US Office of Management and budget issued an Advanced Notice of Proposed Rule Making (ANPRM) to alter the federal regulations governing IRBs (45-CFR-46 – the so-called “Common Rule”). [15], [16]. But given that 15 separate federal departments and agencies are involved with the Common Rule, the ultimate fate of such proposals is unclear, and changes, if any, could take years to become effective. CIRB reviews can also be controversial and disputed because of inherent uncertainties in current regulations (e.g., definitions of “minimal risk”), and new technologies and methodologies. Moreover, in most CIRB models, local IRBs can accept, reject, or amend CIRB recommendations. Hence, ultimate control will presumably remain local; and regardless of whether, and to what degree centralization occurs, tensions will doubtlessly continue.

Thus, crucial questions remain of how IRBs see and respond to these increasing complaints and conflicts, and what else, if anything, can be done. Surprisingly, little, if any, research has examined these issues.

In a recent in-depth semi-structured interview study I conducted of views and approaches toward research integrity (RI) among IRB chairs, directors, administrators, and members, issues concerning relationships between IRBs and PIs repeatedly arose. Participants defined “RI” very broadly, and varied in how they viewed and approached RI [17], conflicts of interest [18], CIRBs [19], unaffiliated and nonscientific members [20], research in the developing world [21], and variations between IRBs [22]. Importantly, they varied, too, in how they saw and approached the roles and responsibilities of IRBs and PIs; viewed, interacted with, and responded to PIs and perceived these relationships. These issues are crucial as they can potentially affect the degree to which PIs adhere to IRB regulations and protect study participants as much as possible. The study, since it used qualitative methods, allowed for detailed explorations of these domains.

## Methods

### Ethics Statement

The Columbia University Department of Psychiatry Institutional Review Board approved the study, and participants all gave informed consent. As approved by the IRB, the consent was verbal, not written, since the interviews with this sample of IRB chairs, members, and staff from across the US were conducted over the telephone, rather than in person. I sent all study participants an informed consent information sheet that they read before being interviewed. They then consented to the interview over the phone, prior to initiating the interview; and I then documented their consent.

### Study Design and Procedures

As described elsewhere [17]–[22], I conducted in-depth telephone interviews of 2 hours each with 46 chairs, directors, administrators, and members. I contacted the leadership of 60 IRBs around the country, representing every fourth one in the list of the top 240 institutions by NIH funding, and interviewed IRB leaders from 34 of these institutions (response rate = 55%). At times, I interviewed both a chair/director and an administrator from an institution (e.g., if the chair thought the administrator might be better positioned to answer certain questions). Thus, from these 34 institutions, I interviewed in all 39 chairs/directors and administrators. The institutions range in location, size, and public/private status. Sampling IRBs from a wide range of institutions allows for illumination of the roles of different social and institutional contexts concerning these issues. I also asked half of these leaders (every other one whom I interviewed on the list by amount of NIH funding [n = 17]) to distribute information about the study to members of their IRBs, in order to recruit 1 member of each of these IRBs to be interviewed as well. Thus, in addition to the 39 chairs/directors and administrators, I interviewed 7 other members (6 regular members and 1 nonscientific/unaffiliated member), yielding a response rate of 41.2% (7/17).

As shown on Table 1, the 46 individuals include 28 chairs/co-chairs; 1 IRB director; 10 administrators (including 2 directors of compliance offices); and 7 members, and they varied in gender, ethnicity, and location.

**Table 1:** Subject Characteristics.

	Total	% (N = 46)
**Type of IRB Staff**		
Chairs/Co-Chairs	28	60.87%
Directors	1	2.17%
Administrators	10	21.74%
Members	7	15.22%
**Gender**		
Male	27	58.70%
Female	19	41.30%
**Institution Rank**		
1–50	13	28.26%
51–100	13	28.26%
101–150	7	15.22%
151–200	1	2.17%
201–250	12	26.09%
**State vs. Private**		
State	19	41.30%
Private	27	58.70%
**Region**		
Northeast	21	45.65%
Midwest	6	13.04%
West	13	28.26%
South	6	13.04%
**Total # of Institutions Represented**	**34**	

The interview explored participants' views of RI, broadly defined, and IRB responses and factors involved in decisions, but elucidated many other, wider issues that emerged regarding IRBs' interactions and relationships with researchers. Several relevant portions of the interview guide appear in Appendix S1. From a theoretical perspective, Geertz [23] has advocated exploring people's lives, decisions, and milieu by trying to grasp their own experiences, through their own words and perspectives to obtain a “thick description.”

In the methods, I have adapted elements from Grounded Theory [24]. This approach is thus informed by techniques of “constant comparison” in which data from different contexts are compared for similarities and differences, to see if they suggest hypotheses. This “constant comparison” yields new analytic categories and questions, and checks them for reasonableness. During the ongoing process of in-depth interviewing, I examined how participants resemble or differ from each other, and the social, cultural, and medical contexts and factors that contribute to variations. Grounded theory involves deductive as well as inductive thinking, building inductively from the data to an understanding of themes and patterns within the data, and deductively, drawing on frameworks from prior research and theories.

I drafted the questionnaire, drawing on prior research I conducted and published studies. Transcriptions and initial analyses of interviews occurred during the period in which the interviews were being conducted, enhancing validity, and these analyses helped shape subsequent interviews.

After the full set of interviews was completed, subsequent analyses were conducted in two phases, by a trained research assistant (RA) and myself. In phase I, we independently examined a subset of interviews to assess factors that shaped participants' experiences, identifying categories of recurrent themes and issues that were subsequently given codes. We read each interview, systematically coding blocks of text to assign “core” codes or categories (e.g., instances of tensions with PIs; beliefs that IRBs had power, or did not have power). While reading the interviews, a topic name (or code) was inserted beside each excerpt of the interview to indicate the themes being discussed. We then worked together to reconcile these independently developed coding schemes into a single scheme. We then prepared a coding manual, defining each code and examining areas of disagreement until reaching consensus between them. New themes that did not fit into the original coding framework were discussed, and modifications were made in the manual when deemed appropriate.

In phase II of the analysis, we then independently content analyzed the data to identify the principal subcategories, and ranges of variation within each of the core codes. The sub-themes identified by each coder were reconciled into a single set of “secondary” codes and an elaborated set of core codes. These codes assess subcategories and other situational and social factors. Such subcategories included, for example, specific reasons why IRBs were thought to have power or not have power, and types of tensions with PIs (e.g., related to PIs' misperceptions of IRBs).

Codes and sub-codes were then used in analysis of all of the interviews. To maximize coding reliability, two coders analyzed all interviews. Where necessary, multiple codes were used. The coders assessed similarities and differences between participants, examining categories that emerged, ranges of variation within categories, and variables that may be involved.

**Figure 1:**
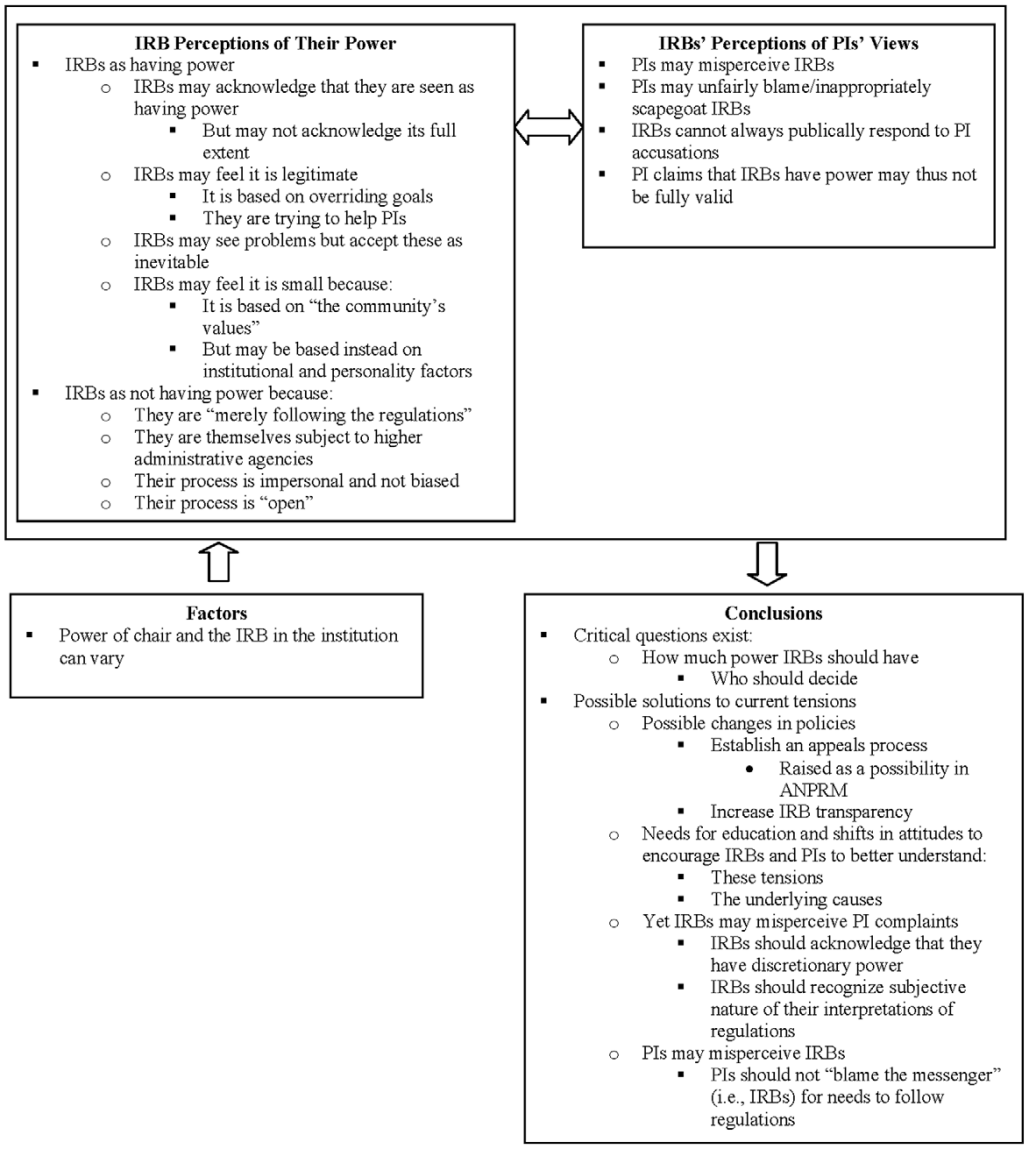
Themes concerning differing views of power and PI complaints among IRBs.

We examined areas of disagreement through closer analysis until consensus was reached through discussion. Overall, disagreements were minimal and generally concerned only subtle refinements of sub-codings, at time necessitating use of dual codes, and were in no cases significant. We checked regularly for consistency and accuracy in ratings by comparing earlier and later coded excerpts. Figure 1 reflects the codes and sub-codes used.

To ensure that the coding schemes established for the core codes and secondary codes are both valid (i.e., well grounded in the data and supportable) and reliable (i.e., consistent in meaning), they were systematically developed and well-documented.

## Results

As summarized in Figure 1, and described more briefly below, many interviewees recognized that they may be seen by PIs as having power; but these interviewees generally thought that they did not have it, or that it was minimal or justified, because they were “merely following the regulations,” had an “open,” impersonal, and unbiased process, and were themselves subject to higher administrative agencies. To the degree that they do have power, IRBs appear to feel that it is legitimate because it is based on “the community's values” and overriding goals. Yet IRBs may misperceive PI complaints.

Several factors may shape these views, including the political position of the chair and the IRB.

### Differing Views of the Nature and Causes of PI Complaints

#### IRBs as Having Power

Interviewees all acknowledged tensions between themselves and researchers, but suggested that they and researchers often saw the nature and causes of these conflicts very differently. At times, interviewees described how they and PIs disagree in how they seem themselves and each other in critical ways, exacerbating tensions.

Several interviewees acknowledged that their IRB had power, but may focus on only particular aspects of it. As one member said,

My IRB is pretty darn powerful. I've been amazed at how many consent forms they will actually send back. We're really hard on the inform consent form. **IRB41**

But the basis of this statement – that the IRB can force changes in consent forms – refers to only one, relatively small potential manifestation of IRBs' potency.

Some interviewees saw their IRBs as having power, but felt that it was legitimate and deserved, even if tensions ensued. A former chair reported,

We respected the PIs as scientists, but they are going to have to do things *our way*. In two or three instances, they thought, “Who the hell are you…to tell me that?” **IRB7**

Other IRB chairs agree they have power, but feel that it's warranted because of overriding goals – protecting subjects and optimizing the quality of protocols.

PIs think the IRB has a lot of power because they can't proceed without approval. *But we're there to help you get your protocol up to snuff*. And the consent form and all the elements of the consent are now templated on the website. PIs just have to fill things out. **IRB27**

But definitions of “up to snuff,” and whether this term refers to the science or the ethics involved, can vary. This chair also suggests that the straightforward nature of these forms means that the IRB does not have power – again seemingly misapprehending PI concerns.

IRBs may dismiss the notion that they have power because they see themselves as helping PIs – e.g., navigating the regulations.

The IRB has power in the sense that we can tell someone they can't do a study, but we've never done that. We can certainly tell them that they need to do it in a certain way. So the IRB has certain power, but on the other hand, we're just trying to help the PI navigate through – to do good research, but to do it in a way that navigates the federal rules appropriately. **IRB19**

Yet she is a social science IRB chair who reviews almost all minimal risk research. In contrast, IRBs that review more invasive research may be more likely to alter or block studies in more fundamental ways.

Chairs may agree they have power, but feel it is legitimate because it is based on “community standards.” However, discrepancies between IRBs may reflect differences in institutional histories (e.g., having been audited and/or “shut down” by OHRP) and in personalities (e.g., a chair being “nitpicky” *vs.* “user-friendly”), more than variations in community values [22]. The absence of an external appeals process can bolster such authority, facilitating subjectivity.

Others feel that they have power, but only a small amount, though some realize that scientists may disagree. “I believe the IRB has power, but not too much power. But scientists would probably disagree with that. We have the power to stop a protocol.” (**IRB35**)

In interacting with PIs, some interviewees recognized that they may be seen by PIs as harsh and potentially insensitive. IRBs may “grill” PIs, making them nervous, yet these committees may seek to justify these approaches.

We have a little bit of a reputation of grilling the investigators when they come in, and I think they're a little bit nervous when they walk in. But I don't think that's necessarily bad, because I don't think everybody should feel like it's a cake walk – that everything's going to get passed. **IRB41**

At times, IRBs recognized that PIs may, as a result of the IRB, decide not to pursue certain studies because of fears of rejection from the IRB. IRBs may see this trend as unfortunate, but nonetheless maintain it, seeing it as inevitable under the current system.

Some faculty have learned to become strategic about projects, to avoid IRB review, and don't study vulnerable populations. But then people don't study children. For instance, one couldn't do a naturalistic study of bullying in the schoolyard. **IRB22**

Several factors may affect the extent of an IRB's power. For instance, the IRB chair may also be a department head, who thus has additional clout. Such institutional authority appears very important – i.e., in backing the IRB.

…our IRB chair is also the department chair. So we are dealing with someone who is in a position of authority *outside* of the IRB, so as a result of his position we get less flack from PIs. **IRB9**

#### IRBs Not Having Power

Yet IRB chairs and members may see themselves as *not* having power, or as having it, but feeling it's justified.

Several interviewees said they did not understand these claims that they possess power. Several types of reasons arose in defense of these views. IRBs may think that they lack power because they are “merely following the regulations.”

I don't really get it when the FDA says the IRB has so much power. It's only through regulations. And if things are designed according to the regulations, then it's not a matter of power. **IRB13**

Yet “merely following the regulations” does not necessarily preclude the existence of power. Given discrepancies in how committees interpret and apply these regulations, IRBs appear to have discretionary power – i.e., abilities to interpret and apply regulations differently.

IRBs may think they lack power, too, because they themselves feel besieged, disrespected, and disliked. IRBs can themselves be audited and criticized by federal agencies. (“Where are IRBs very powerful, and not the objects of scorn?” **IRB13)**

Additionally, IRBs may feel that they lack power because they follow logical, fairly impersonal processes, and are not biased against any particular researchers. The interviewee above continued,

We don't have the power to say, “That doctor was really rude to us last year: he complained at the faculty meeting about how the IRB was slow, and lost studies. OK, we'll fix him! We'll put him on the July agenda.” **IRB13**

But in suggesting that IRBs lack power because they do not use their role to pursue illegitimate, personal vendettas, this administrator may in fact be creating a “straw dog,” unfairly characterizing PI criticisms of IRBs.

IRBs may also dismiss claims that they have power because their processes are transparent, and IRBs are “open.” This interviewee added, further explaining her views,

We're real visible here. At some hospitals, researchers submit stuff to the IRBs, and it's a closed door. They can't enter the sanctum. At *our* IRB, all the staff are available all of the time without appointment. You just come in. PIs know me. **IRB13**

She sees her IRB as “open” and thus not powerful, suggesting several issues concerning the definition, perception, and legitimacy of power. Yet openness can reduce power, but not necessarily eliminate it.

#### IRBs' Perceptions of PIs' Views

Yet IRBs also see PIs misunderstanding IRBs. PIs may unfairly hold the IRB responsible for other difficulties, unjustifiably blaming the IRB after turning in late, incomplete, or sloppy forms, exacerbating tensions. As one administrator said:

People want to blame something. If a PI can't start a study – a sponsor was unable to, or decided not to use the site – the IRB is always a good place to blame: “*If it hadn't been for the IRB!*…We lost that study because the IRB didn't act quickly enough.” OK, well, let's see the protocol: it says it was issued last July, and it's coming over here in February…We can be the source of all joy or all sorrow, depending on how their grant funding worked out. **IRB13**

To a degree, PIs may thus at times “blame the messenger” – i.e., the IRB – for needs to follow federal regulations. Yet the ability to thwart another's desires can be seen as representing power. Power may thus partly be in the eyes of the beholder, and two parties may disagree. Nonetheless, IRBs emerge here as, in effect, “caught in the middle” between federal regulations and agencies on the one hand serving as their local interpreter, enforcer, and “face,” and local PIs on the other.

Some felt PIs may unfairly blame IRBs as an easy target. PIs may fault these committees for delays and complain to institutional leaders who then pressure IRBs or send protocols to for-profit CIRBs. One chair, at an institution that recently began also using such a CIRB, said,

A lot of the political pressure, or frustration with our whole review system was directed at the IRB when, in reality, I think we were doing pretty good with our turnarounds. Other steps, or reviews – the scientific advisory committee, and the grants and contracts office – were becoming problematic. Maybe we can turn it around a little quicker. But I don't think studies are necessarily starting any quicker – because of budget issues, or the hospital representative has a problem with an injury compensation statement that we're just fine with. It was a PR problem. Many times the IRB was the fall guy. It's easy and quick to say, “It's *their* fault.” Rather than really doing a process analysis and figuring out where all the other delays are, it was easier to say, “Let's take this to WIRB [Western IRB – an independent, for-profit IRB] and get our turnaround times approved.” **IRB6**

An IRB's position may be complicated by the fact that it cannot publicly respond if PIs vocally fault the committee within an institution. Such limited communication can aggravate strains.

People assume the IRB is the big-evil-regulating-snooty-bureaucracy, and that the researcher did everything right. But we can't say, “The PI might have said *this*, but *here's* the truth…” **IRB22**

Chairs may also misperceive or underappreciate PI complaints, as well as underlying tensions, and potential responses. “PIs are primarily concerned with how *quickly* a project can be approved with minimal comments. So, IRBs need additional staff to absorb the workload” (**IRB9**). Yet IRB attitudes, not only resources, may need to change.

## Discussion

This study, the first to explore how IRBs view power relationships with PIs, suggests that IRBs and researchers may differ in how they view their respective roles and relationships, exacerbating tensions between them. While PIs may see IRBs as having power, IRBs themselves can disagree, and deny that they have it, or seek to justify it.

One can argue that these tensions are inevitable and unavoidable, given the different institutional roles of PIs and IRBs, and may even be desirable to some degree. After all, if relationships are too cozy, human subjects may not be adequately protected.

The key questions, though, are not whether such power should exist, but how much. Specifically, these data highlight questions of how much power IRBs should have and in what ways, who should decide, what the costs are of these committees having too much power, and what checks and balances should exist. Clearly, disagreements can occur here. IRB chairs may know that they are seen as “obstructionistic” by PIs, but differ in how much they are troubled by, or try to alter these perceptions.

IRBs appear to try to justify their power, arguing that it helps PIs and human subjects, though that claim may not be based on empirical evidence, and may actually cause harms that the IRB may not sufficiently acknowledge or weigh, delaying or impeding valuable research.

These conflicting views of IRB power may partly reflect larger social structures and tensions within complex, hierarchical academic medical institutions. Yet both sides can, ideally, work to ameliorate these conflicts.

IRBs' power may be legitimate, but discretionary and subjective. An IRB's ability to interpret and apply regulations as it thinks best confers an important degree of authority. A committee can follow specified processes, but still interpret regulations subjectively.

While prior research has explored researcher views of the “fairness” of IRBs [3], [12], the present data highlight another aspect of these views – that IRBs have power that can incur resentment.

Much of IRB work occurs behind closed doors, which can aggravate these tensions. Minutes are not publicly accessible, but arguably attempts should be make these available, at least in part, to whatever degree may be reasonable and possible. IRBs keep minutes private, along with all correspondence and decisions (except to the PI involved). Yet increasing transparency could potentially help improve perceptions of IRBs among PIs. Given concerns about proprietary information, redaction of details at any institution may be hard for certain studies, particularly those that are industry-funded. Yet transparency may not be as difficult for many other, non-industry funded protocols. IRBs could, for instance, post examples, with details redacted, of the types of concerns they have had about issues that arise in various protocols. Such an approach could yield many benefits. Yet IRBs may themselves prefer the lack of transparency, as it may reduce questions about their processes and decisions – which presumably is not the intended goal of the current practice of non-transparency. These interviews thus highlight key questions of how much lack of transparency is, or should be permitted.

Educational efforts and shifts in attitudes can thus be helpful. IRBs and PIs should both be encouraged to strive to understand more clearly the nature of tensions between them, the underlying causes, and possible ways to address these. IRBs should recognize these subjective elements more fully, and weigh this awareness more in decisions. Many IRBs try to respond to PIs' complaints, improve relationships, and “not be the ethics police,” but vary widely in how.

From Aristotle [25] to Madison and Hamilton in the Federalist Papers [26], theorists have argued that power can play vital roles in political and social structures, but can be used well or poorly. So, too, IRBs will invariably, it seems, have a modicum of authority and thus power, but can use it in ways that are more or less justified and effective. More recently, “discretionary power” has been described with regard to police, who can choose when and how to employ it [27]. Yet unlike the police and many other entities with power in a democratic civil society, with IRBs, no appeals process exists. Citizens can readily file complaints about police officers, and have these grievances addressed by higher administrative authorities that can correct or remedy perceived wrongs. But for researchers, no such processes have yet been systematically established.

IRBs should realize that the absence of an appeals process gives them de facto considerable power. To ignore this fact can exacerbate tensions between IRBs and PIs, while increased acknowledgement of this perceived power can help IRBs facilitate interactions with PIs, and thus in the end best help protect human subjects.

Concomitantly, PIs may also unfairly “blame the messenger,” resisting federal regulations, exacerbating conflicts [17]. Researchers should realize that IRBs, while subjectively implementing these regulations, are in fact also constrained in many ways by these policies, and fears of governmental audits, and generally appear to be trying their best.

Needs thus exist, too, to further enhance education about IRB regulations and the underlying principles and processes of review among medical faculty and trainees, to help reduce these tensions in the future. While existing educational mandates may foster resentment among some researchers, such education can nonetheless be extremely important. Requirements among physicians for board certification, and continuing medical education may also be perceived burdensome, but have been accepted as important to optimize the standards and quality of care. Further questions about research ethics could be included, for instance, in board examinations. Improved understanding of the broader context of IRBs and IRB regulations can ultimately enhance compliance and protection of human subjects.

A critic might dismiss these educational and attitudinal efforts as unimportant, or as too aspirational to be attainable through policy. However, increased awareness of these issues through discussions in peer-reviewed journals, at academic meetings, and in other fora can heighten awareness of these issues in ways that can enhance the system. Indeed, policy changes, as now being recommended through the ANPRM, may have unintended consequences, and may not be able to wholly solve all of the tensions and differing perspectives that these interviewees describe. Hence, such education and attitudinal shifts can be vital.

These data also have several critical implications for policy, and can aid discussions concerning possible changes in policies that are raised in the ANPRM. For instance, these data suggest needs for an appeals process. The ANPRM asks whether institutions should all be required to provide an appeals mechanism, and if so, what type. The present data are thus important in highlighting reasons why such an appeals process can be advantageous. These data, highlighting the role of IRB power, suggest that such an appeal not be made to the same IRB that made the initial decision, but rather to an independent body. The details involved would have to be determined. For example, as one possibility, the researcher could send a memo documenting the reasons for his or her appeal, to which the IRB could respond. Both documents could then be submitted as an appeal (e.g., to another IRB at that or another institution).

In addition, having one committee serve as the IRB of record in multi-site studies, as proposed in the ANPRM, could potentially decrease the power of other, local IRBs. This committee of record would, however, then assume considerable clout. IRBs having to report to OHRP if they deviate from aspects of the proposed new regulations, as raised as a possibility in the ANPRM, can also prompt IRBs to curb any idiosyncrasies that may exist, and may serve to create checks and balances, and decrease these committees' power.

These data have actual implications for research as well. Research is critical to explore more fully how IRBs and PIs each view and respond to these tensions, what factors are involved, and what interventions might help. Future studies can probe these areas further with larger samples, examining, for instance, how factors (such as federal audits at an institution) may affect views of IRB power, and whether differences in these views correlate with differing perceptions and practices among PIs. For instance, those chairs who see themselves as having power, but try to minimize its use, may result in PIs who feel less resentful of IRBs, value research ethics more, follow regulations more closely, and violate research integrity less.

These findings have several potential limitations. These interviews explored subjects' views at present and in the past, but not prospectively over time, to examine possible changes. This study did not interview researchers as well at these institutions, but future studies can do so as well. Further studies can collect and analyze these additional sources of data. The response rate among regular members was not as high as among chairs and administrators, since I did not contact regular members directly, but relied on chairs and administrators disseminating information about the study to these regular members, and then having these regular members contact me. I do not know whether the chairs and administrators in fact distributed this information, and if so, did so once or more than once. Hence, the response rate was lower. These findings are also based on in-depth interviews with individual IRB chairs and members, and did not include direct observations of IRBs as a whole, or examination of written IRB records. Future research can, however, observe IRBs. Yet, such added data may be hard to procure since, anecdotally, IRBs have generally required researchers to obtain consent from all IRB members and the relevant PIs, and protocol funders. This study is qualitative, and thus is designed to reveal, in many ways that quantitative data cannot, beliefs, attitudes, views, and relationships between these phenomena, generating research questions and hypotheses that future investigations can examine in further detail among larger samples, employing both qualitative and quantitative approaches. Qualitative research is not designed to measure responses quantitatively. But future investigations can address these questions.

These points may be controversial, but as science, and hence needs for adequate subject protection, further advance, addressing these conflicts and their underlying sources is essential.

## Supporting Information

Appendix S1 **Sample questions from semi-structured interview.** *Note: Additional follow-up questions were asked, as appropriate, with each participant.*
(DOC)

## References

[1] Burris S (2008). Regulatory innovation in the governance of human subjects research: A cautionary tale and some modest proposals.. Regulation Governance.

[2] Burris S, Moss K (2006). U. S. health researchers review their ethics review boards: A qualitative study.. J Empir Res Hum Res Ethics.

[3] Keith-Spiegel P, Koocher GP, Tabachnick B (2006). What scientists want from their research ethics committees.. J Empir Res Hum Res Ethics.

[4] Koerner AF (2005). Communication scholars' communication and relationship with their IRBs.. J Appl Commun Res.

[5] Gunsalus CK, Bruner EM, Burbules NC, Dash L, Finkin M (2006). Mission creep in the IRB world.. Science.

[6] Bell J, Whiton J, Connelly S (1998). Final report: Evaluation of NIH implementation of section 491 of the public health service act, mandating a program of protection for research subjects.

[7] McWilliams R, Hoover-Fong J, Hamosh A, Beck S, Beaty T (2003). Problematic variation in local institutional review of a multicenter genetic epidemiology study.. JAMA.

[8] Dziak K, Anderson R, Sevick MA, Weisman CS, Levine DW (2005). Variations among institutional review boards in a multisite health services research study.. Health Serv Res.

[9] Greene SM, Geiger AM (2006). A review finds that multicenter studies face substantial challenges but strategies exist to achieve Institutional Review Board approval.. J Clin Epidemiol.

[10] Fleischman AR (2005). Regulating research with human subjects: Is the system broken?. Trans Am Clin Climatol Assoc.

[11] Hamburger P (2004). The new censorship: Institutional Review Boards.. Supreme Court Rev.

[12] Keith-Spiegel P, Koocher GP (2005). The IRB paradox: Could the protectors also encourage deceit?. Ethics Behav.

[13] Menikoff J (2010). The paradoxical problem with multiple-IRB review.. New Engl J Med.

[14] Ahmed AH, Nicholson KG (1996). Delays and diversity in the practice of local research ethics committees.. J Med Ethics.

[15] Department of Health and Human Services (2011). “Human subjects research protections: enhancing protections for research subjects and reducing burden, delay, and ambiguity for investigators.”. Federal Register.

[16] Emanuel EJ, Menikoff J (2011). Reforming the regulations governing research with human subjects.. New Engl J Med.

[17] Klitzman R (2011). Views and experiences of IRBs concerning research integrity.. J Law Med Ethics.

[18] Klitzman R (2011). “Members of the same club”: Challenges and decisions faced by US IRBs in identifying and managing conflicts of interest.. PLoS ONE.

[19] Klitzman R (2011). How local IRBs view central IRBs in the US.. BMC Med Ethics.

[20] Klitzman R (2011). ‘Community’ IRB members: Who are they, what do they do, and do they represent anyone?. Acad Med.

[21] Klitzman R (2011). US IRBs confronting research in the developing world.. Dev World Bioeth.

[22] Klitzman R (2011). The myth of community differences as the cause of discrepancies between IRBs.. Amer J Bioeth.

[23] Geertz C (1973). Interpretation of Cultures: Selected Essays.

[24] Strauss A, Corbin J (1990). Basics of qualitative research: Techniques and procedures for developing grounded theory.

[25] Aristotle (Saunders TJ, ed.) (1981). The Politics.

[26] Hamilton A, Madison J (2010). The Federalist Papers.. http://www.foundingfathers.info/federalistpapers/.

[27] Smith DA, Visher CA, Davidson LA (1984). Equity and discretionary justice: The influence of race on police arrest decisions.. J Crim Law Criminol.

