# Supplementary material for: The Ethics Police?: IRBs' Views Concerning Their Power
Source: PLoS One. 2011 Dec 13;6(12):e28773. doi: 10.1371/journal.pone.0028773 (PMC3236766; doi:10.1371/journal.pone.0028773)
Supplement: Appendix S1 — Sample questions from semi-structured interview. Note: Additional follow-up questions were asked, as appropriate, with each participant. (DOC) [file pone.0028773.s001.doc]

**Appendix S1: Sample Questions from Semi-Structured Interview***

- How do you define research integrity (RI)? What has been the most difficult case concerning RI that you have faced? What kinds of issues arose? Do you think IRBs and PIs view RI differently or apply RI standards differently, and if so, how? Have you seen problems in researcher non-compliance with IRB regulations or mandates? If so, what kinds of problems? What happened?
- What are the barriers and facilitators in IRBs monitoring and addressing RI problems among PIs? Do you perceive any gray areas or problems weighing issues about RI among PIs? If so, what? How do you address these?
- Is your IRB more cautious about some researchers than others? Why? In general, do PIs treat your IRB with respect?
- Has your IRB discussed sanctions against PIs?
- What do you think makes an IRB work well or not in monitoring and responding to RI among PIs?
- What kinds of conflicts, if any, has your IRB faced with your institution? Why do these occur?
- Do you have any other thoughts about these issues?

**Note: Additional follow-up questions were asked, as appropriate, with each participant.*
